# Distribution and Incorporation of Extracellular Vesicles into Chondrocytes and Synoviocytes

**DOI:** 10.3390/ijms252211942

**Published:** 2024-11-06

**Authors:** Takashi Ohtsuki, Ikumi Sato, Ren Takashita, Shintaro Kodama, Kentaro Ikemura, Gabriel Opoku, Shogo Watanabe, Takayuki Furumatsu, Hiroshi Yamada, Mitsuru Ando, Kazunari Akiyoshi, Keiichiro Nishida, Satoshi Hirohata

**Affiliations:** 1 Department of Medical Technology, Graduate School of Health Sciences, Okayama University, 2-5-1, Shikata-cho, Kita-ku, Okayama 700-8558, Japan; sohmaohtsuki@yahoo.co.jp (T.O.); ikm-sato@s.okayama-u.ac.jp (I.S.); pn229drz@s.okayama-u.ac.jp (K.I.); gabbyjnr53@gmail.com (G.O.); watanabe1224@okayama-u.ac.jp (S.W.); 2 Department of Orthopedic Surgery, Dentistry and Pharmaceutical Sciences, Okayama University Graduate School of Medicine, 2-5-1, Shikata-cho, Kita-ku, Okayama 700-8558, Japan; takamatino@gmail.com (T.F.); knishida@md.okayama-u.ac.jp (K.N.); 3 Department of Neuroscience, Dentistry and Pharmaceutical Sciences, Okayama University Graduate School of Medicine, 2-5-1, Shikata-cho, Kita-ku, Okayama 700-8558, Japan; hiroyama@md.okayama-u.ac.jp; 4 Laboratory of Biomaterials, Institute for Life and Medical Sciences, Kyoto University, Kawahara-cho Shogoin, Sakyo-ku, Kyoto 606-8507, Japan; ando@infront.kyoto-u.ac.jp; 5 Department of Immunology, Graduate School of Medicine, Kyoto University, Yoshida-Konoe-cho, Sakyo-ku, Kyoto 606-8501, Japan; akiyoshi.kazunari.2e@kyoto-u.ac.jp

**Keywords:** extracellular vesicles (EVs), chondrocytes, synoviocytes, osteoarthritis (OA)

## Abstract

Osteoarthritis (OA) is a chronic disease affecting over 500 million people worldwide. As the population ages and obesity rates rise, the societal burden of OA is increasing. Pro-inflammatory cytokines, particularly interleukin-1β, are implicated in the pathogenesis of OA. Recent studies suggest that crosstalk between cartilage and synovium contributes to OA development, but the mechanisms remain unclear. Extracellular vesicles (EVs) were purified from cell culture-conditioned medium via ultracentrifugation and confirmed using transmission electron microscopy, nanoparticle tracking analysis, and western blotting. We demonstrated that EVs were taken up by human synoviocytes and chondrocytes in vitro, while in vivo experiments revealed that fluorescent-labelled EVs injected into mouse joints were incorporated into chondrocytes and synoviocytes. EV uptake was significantly inhibited by dynamin-mediated endocytosis inhibitors, indicating that endocytosis plays a major role in this process. Additionally, co-culture experiments with HEK-293 cells expressing red fluorescent protein (RFP)-tagged CD9 and the chondrocytic cell line OUMS-27 confirmed the transfer of RFP-positive EVs across a 600-nm but not a 30-nm filter. These findings suggest that EVs from chondrocytes are released into joint fluid and taken up by cells within the cartilage, potentially facilitating communication between cartilage and synovium. The results underscore the importance of EVs in OA pathophysiology.

## 1. Introduction

Osteoarthritis (OA) is the most prevalent chronic joint disorder, leading to joint deterioration, pain, stiffness, and impaired function [1,2,3]. The primary pathological features of OA include cartilage destruction and osteophyte formation, both of which are largely driven by inflammation. In OA, elevated levels of pro-inflammatory cytokines in the synovial fluid contribute to disease progression by accelerating extracellular matrix (ECM) degradation [4,5,6]. The articular cartilage ECM is primarily composed of type II collagen, aggrecan, and hyaluronic acid (HA), which provide the tissue with its mechanical strength and elasticity [7,8]. However, during OA progression, the breakdown of these ECM components is primarily driven by matrix-degrading enzymes such as matrix metalloproteinases (MMPs) and aggrecanases (ADAMTS) [9,10].

Aggrecan, a key component of the ECM, plays a crucial role in retaining water within the cartilage matrix, ensuring tissue elasticity and durability. The degradation of aggrecan, caused by the overactivity of MMPs and ADAMTS, results in a loss of these critical properties and contributes to cartilage destruction. Prior research has shown that the activity of these enzymes can be modulated by various factors, including mechanical stress and the addition of high-molecular-weight HA [11,12]. In particular, cytokines such as interleukin-1β and tumor necrosis factor-alpha have been shown to induce the expression of MMP13, ADAMTS4, and ADAMTS9 in chondrocytes, further accelerating cartilage degradation.

Recent studies have reported the role of extracellular vesicles (EVs) not only in maintaining joint homeostasis but also in the progression of OA by modulating interleukin-1β [13,14]. EVs are membranous vesicles secreted by cells that carry bioactive molecules, such as proteins, lipids, and nucleic acids, to recipient cells. These vesicles play a vital role in mediating cell-to-cell communication in both local and distant tissues, influencing cellular behavior and function [15,16]. In OA, synovitis and cartilage degradation have been attracting attention, and the role of EVs in cell-to-cell communication is promising.

However, the specific mechanisms by which EVs contribute to the progression of OA are yet to be clarified. Therefore, in this study, we aimed to investigate the role of EVs in the crosstalk between chondrocytes and synoviocytes. By examining the transfer of EVs between these cells, we sought to elucidate how EV-mediated communication might drive synovial inflammation and cartilage degradation in OA.

## 2. Results

### 2.1. Characterization of EVs

**Figure 1:**
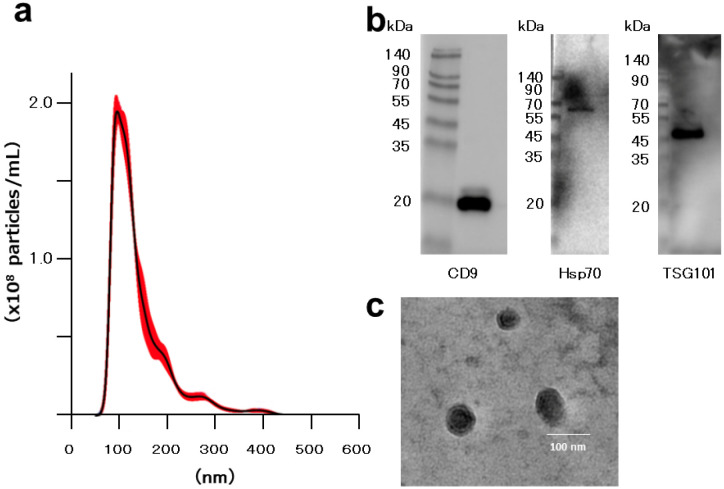
Characterization of EVs derived from OUMS-27. (**a**) Size distribution of EVs is shown. EVs ranged from 50 to 500 nm. (**b**) Western blot analysis of EV surface marker expression (CD9, Hsp70, and TSG101 chosen from the exosome panel) is shown. (**c**) Representative transmission electron microscopy (TEM) images of EVs (scale bar  =  100 nm). The black line indicates the mean and the red area indicates the standard deviation of five measurements of the same samples.

We characterized the isolated EVs by analyzing their size, morphology, and surface markers. EVs were isolated from 100 mL of OUMS-27 conditioned medium using ultracentrifugation and resuspended in 0.5 mL of phosphate-buffered saline (PBS). Nanoparticle tracking analysis (NTA) revealed that the EVs ranged in size from approximately 50 to 450 nm in diameter, which is consistent with typical EV dimensions (Figure 1a). From 1.0 × 10^6^ OUMS-27 cells, 8.0 × 10^8^ EV particles were produced. Western blotting confirmed the presence of the EV surface marker proteins CD9, Hsp70, and TSG101 in the isolated EVs (Figure 1b). Additionally, transmission electron microscopy (TEM) confirmed the vesicular morphology of the EVs (Figure 1c)

### 2.2. Uptake of PKH67-Labelled EVs

**Figure 2:**
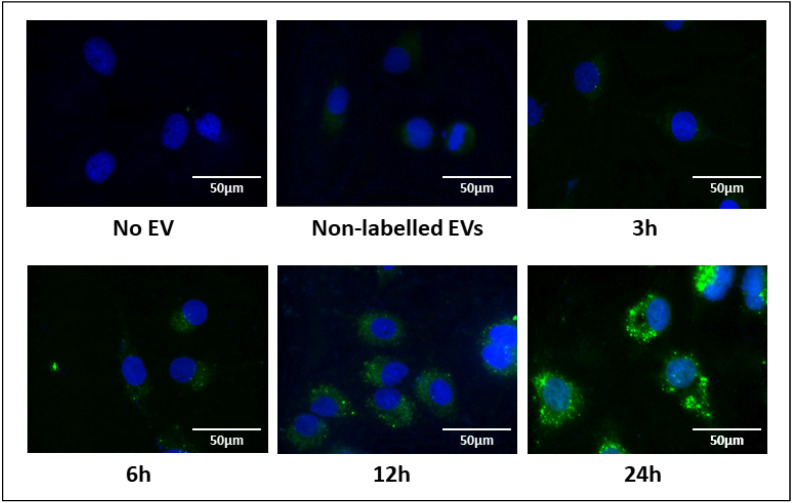
Uptake of EVs in OUMS-27 cells. PKH67-labelled EVs were added to OUMS-27 cell culture medium. Green signals indicate PKH67-labelled EVs and blue refers to nuclei. EVs and nuclei were observed at 3 h, 6 h, 12 h and 24 h by fluorescence microscopy (scale bar  =  50 μm). Green signals indicate PKH67-labelled EVs and blue refers to nuclei.

We next investigated the uptake of PKH67-labelled EVs by OUMS-27 chondrocytes. PKH67-labelled EVs were added to the culture medium, and their uptake by OUMS-27 was monitored over time. EV uptake was faintly visible in the cytoplasm of OUMS-27 cells at 3 h, with an increase in signal intensity at 6 h and 12 h, which peaked at 24 h (Figure 2). No fluorescence signal was observed in negative controls, such as when EVs were not added or non-labelled EVs were added (Figure 2).

### 2.3. Inhibition of EV Uptake by Dynamin Inhibitor

**Figure 3:**
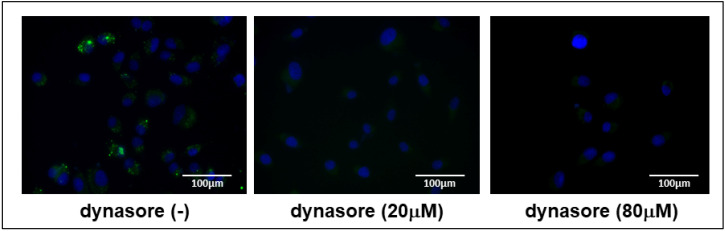
Inhibition of EV uptake by dynasore (dynamin GTPase inhibitor) in OUMS-27. OUMS-27 cells were pretreated with (20 μM and 80 μM) or without dynasore for 24 h, then PKH67-EVs were added to the OUMS-27 cell culture medium (scale bar  =  100 μm). Green signals indicate PKH67-labelled EVs and blue refers to nuclei.

Dynasore, a non-competitive inhibitor, is a membrane permeable small molecule, which decreases endocytosis, cell spreading, and cell attachment. The reagent has been widely used for analyzing dynamin-dependent endocytosis [17]. To elucidate the mechanism of EV uptake in OUMS-27 cells, we examined the effect of dynasore (20 μM and 80 μM), a dynamin GTPase inhibitor, on the uptake process. PKH67-labelled EVs were added to the culture medium with or without dynasore pretreatment. Pretreatment with dynasore considerably inhibited EV uptake compared to vehicle-treated controls (Figure 3), suggesting that EV uptake is mediated through dynamin-dependent endocytosis in OUMS-27 cells.

### 2.4. Transfer and Uptake of Red Fluorescent Protein (RFP)-Tagged CD9 EVs from HEK-293 Cells

**Figure 4:**
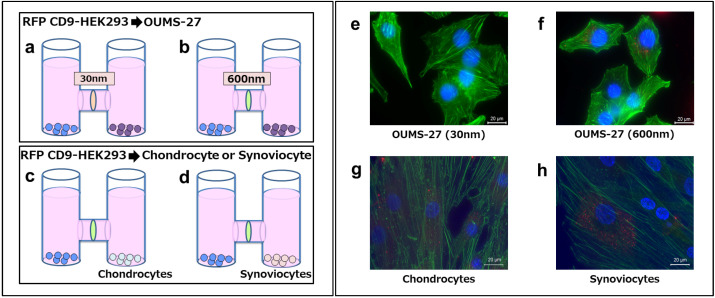
RFP-tagged CD9 expressing EVs transfer. In the co-culture system, RFP-CD9 HEK-293 cells, OUMS-27 cells, and chondrocytes and synoviocytes from patients with OA were seeded in different culture chambers joined horizontally with sharing medium but separated by filters. RFP-CD9 HEK-293 and OUMS-27 cells were co-cultured with a 30-nm filter (**a**) or a 600-nm filter (**b**). RFP-CD9 HEK-293 cells and chondrocytes (**c**) and synoviocytes (**d**) of patients with OA were co-cultured with a 600-nm filter. (**e**) RFP-CD9 signals were rarely observed in OUMS-27 cells with a 30-nm filter, indicating that EVs did not pass through the filter and did not incorporate into OUMS-27 cells. (**f**) RFP-CD9 signals were observed in OUMS-27 cells with the 600-nm filter, indicating that EVs passed through the filter and incorporated into OUMS-27. (**g**) RFP-CD9 signals were observed in the chondrocytes. (**h**) RFP-CD9 signals were observed in the synoviocytes. Red refers to RFP-CD9 of EVs, green refers to filamentous actin (F-actin) and blue refers to nuclei. (scale bar  =  20 µm). (**a**,**b**) Blue color indicates the HEK-293 cells and purple color indicates the OUMS-27 cells. (**c**,**d**) Blue color indicates the HEK-293 cells and light blue color indicates chondrocytes and pink color indicates synoviocytes as indicated below.

We further investigated whether EVs secreted into the culture medium could be incorporated into OUMS-27 cells using a horizontal co-culture system. Human embryonic kidney (HEK-293) cells stably expressing RFP-tagged CD9 were co-cultured with OUMS-27 cells. Using a 600-nm filter, RFP signals originating from HEK-293 cells were detected in OUMS-27 cells (Figure 4f). In contrast, no RFP signal was observed when a 30-nm filter was used (Figure 4e). Additionally, we confirmed the incorporation of RFP-tagged CD9 signals into both cultured chondrocytes and synoviocytes (Figure 4g,h). These results indicate that EVs secreted by HEK-293 cells passed through the 600-nm filter and were subsequently taken up by OUMS-27 cells.

### 2.5. In Vivo Uptake of Labelled EVs in Mouse Joint Tissues

**Figure 5:**
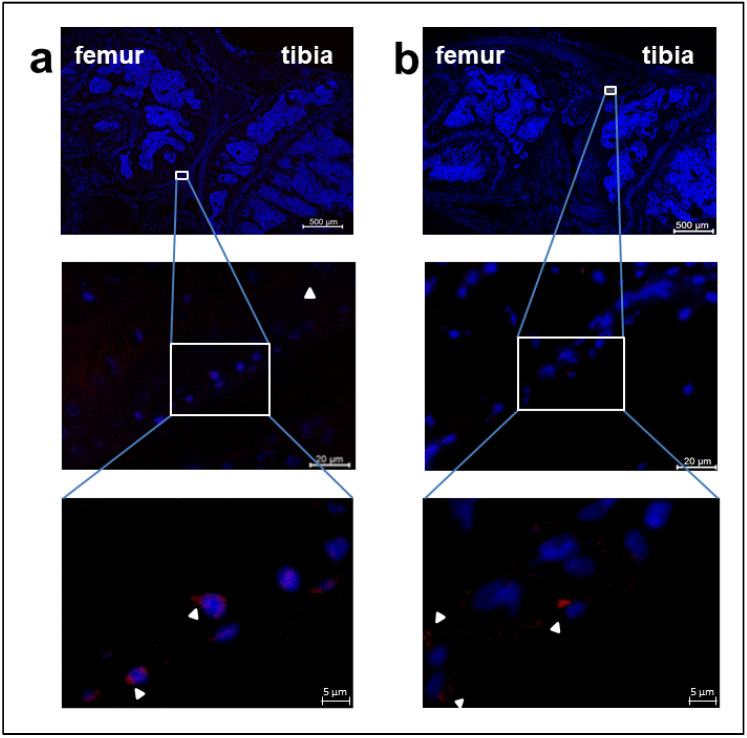
Injection of labelled EVs into the mouse joint cavity and uptake of labelled EVs in mouse joint tissues. Particles (3.0 × 10^8^) of immunofluorescent-labelled EVs were injected into the left hind knee joint of C57BL/6J mice. Mice were sacrificed, and femurs were prepared at 24 h after injection. Frozen sections (5 μm) of joint tissue were made using the Kawamoto’s film method. (**a**) Red fluorescent signals were observed in chondrocytes. (**b**) Red fluorescent signals were observed in synoviocytes. Red refers to Mem Dye-Deep Red labelled EVs and blue refers to nuclei (scale bar  =  500 μm in the top, 20 μm in the middle, 5 μm in the bottom).

Finally, we examined the uptake of Mem Dye-Deep Red -labelled EVs in vivo. Mem Dye-Deep Red labelled EVs (3.0 × 10^8^ particles in 20 µL) were injected into the left knee joint of mice. Fluorescent signals from the labelled EVs were observed in the cytoplasm of chondrocytes and synoviocytes in the joint tissues (Figure 5a,b). In contrast, the right knee joint, which received an injection of non-labelled EVs, exhibited no detectable signals confirming that the observed fluorescence was specific to the labelled EVs at the injection site.

## 3. Discussion

In this study, we examined the uptake of EVs by chondrocytes and synoviocytes using both in vitro and in vivo models, identifying dynamin-mediated endocytosis as a key mechanism. Our findings demonstrate that EVs are taken up by joint cells after being secreted into the culture medium, as well as after intra-articular injections into mouse knee joints. These results emphasize the potential for EVs to function as effective mediators of intercellular communication in joint tissues, which is consistent with the role of EVs in cellular signaling reported elsewhere [18]. Therefore, EVs have potential as a therapeutic tool for managing various conditions. Moreover, disease-associated EVs serve as candidates for targeted inhibition by pharmacological or genetic means [19]. It is clear that a further understanding of EVs is needed for achieving clinical use, and new findings are accumulating in relation to therapeutic aspects such as EV pre-conditioning [20,21,22].

EVs have recently gained attention for their ability to transport a variety of biomolecules, such as DNA, RNA, proteins, and lipids across cells, thereby playing a critical role in maintaining homeostasis and in tissue repair. We first validated the EVs used in this study with standard methods (NTA, TEM, and western blotting analysis), and our EV data was consistent with typical EV characteristics [23]. Our observation that EV uptake by OUMS-27 peaked at 24 h is in line with earlier reports highlighting the capability of EVs to modulate cellular responses and sustain cellular homeostasis [24]. The uptake of EVs by chondrocytes and synoviocytes further underscores their role in joint tissue regulation, offering insights into potential therapeutic applications for joint-related diseases, including OA.

Endocytosis is performed by several agents such as dynamin, glycosylphosphatidylinositol -anchored protein, Arf6, flotillins, and calcium [25]. Clathrin-mediated endocytosis (CME) is ubiquitous in eukaryotic cells. CME is the major mechanism for internalization of macromolecules, proteins, lipids, and EVs. During clathrin-coated vesicle formation in endocytosis, dynamin is recruited to the neck of the forming vesicles, forms a helix, and induces membrane fission by its GTPase activity. We observed that treatment with dynasore [17], a dynamin GTPase inhibitor, significantly inhibited the internalization of PKH67-labelled EVs by OUMS-27 cells. This highlights the role of dynamin-dependent endocytosis for EV internalization in chondrocytes [23], offering insights into potential therapeutic strategies to modulate EV uptake in cartilage.

In our study, we detected RFP-tagged CD9 signals in OUMS-27 cells with a 600-nm but not with a 30-nm filter in a co-culture system. This findings suggests that RFP-tagged CD9 on EVs, primarily exosomes based on their size (30–250 nm), were successfully transferred and taken up by the recipient cells [26,27]. This in vitro system does not represent the complexity of joint tissues. Nevertheless, our results with co-culture system clearly showed that EVs are secreted and diffused, then incorporated into the other cells, indicating that EV can be a tool for cell-cell communication in the synovial fluid.

In previous reports, isolated EVs from various cells were uptaken by recipient cells and changed their characteristics (proliferation, metastasis, invasion, cell survival) and cellular environment (ECM composition) according to their contents (microRNA, nc-RNA, proteins, lipids, polysaccharides) and not by humoral factors (growth factors, cytokines, chemokines) [28]. EVs themselves provide multiple advantages due to their immunomodulatory activity, long circulation half-life, high biocompatibility, transfection efficiency, low immunogenicity, and minimal reversion to virulence [29].

In addition to intrinsic EV properties, this selective transfer of EVs underscores their potential for targeted molecular delivery. EVs have shown promise as therapeutic tools for OA, primarily due to their role in mediating intercellular communication and promoting tissue repair [30]. Additionally, further exploration of EV engineering approaches and culture systems, such as preconditioning with hypoxia or cytokines, surface modifications, and three-dimensional cultures, may enhance their therapeutic efficacy for joint diseases [31,32,33].

Recent studies have reported how EVs are capable of modulating OA progression [34]. Our study confirmed that fluorescent-labelled EVs were taken up by chondrocytes and synoviocytes following intra-articular injection into mouse knee joints. The localization and internalization of the EVs at the injection site indicated a high specificity for joint tissues [35]. Given that articular cartilage is avascular, the injected EVs predominantly remained within the joint and did not migrate to other tissues or organs, including the contralateral knee joint. Although further investigation is required, these results suggest that EV uptake mechanisms may be shared between chondrocytes and synoviocytes, at least in part. It should be noted that our in vivo study was performed in normal mice, and further investigation with an OA mice model is required. Nevertheless, our findings provide valuable insights into the therapeutic potential of EVs for treating joint-related diseases and disorders.

While no specific markers have been identified to distinguish EV uptake by chondrocytes or synoviocytes, the protein profiles of EVs offer valuable information for their potential application in OA treatment. Utilizing omics approaches could provide further insights into the molecular signatures of EVs and enhance our understanding of their roles in cartilage. An important observation in our study was that Mem Dye-Deep Red-labelled EVs injected into the left knee joint of mice remained localized at the injection site, while no signals were detected in the right knee joint, where non-labeled EVs were injected [35]. This supports the notion that locally injected EVs act at the site of administration without migrating to distant tissues, reinforcing their potential for targeted therapeutic applications.

Despite the valuable insights gained from this study on EV uptake mechanisms in joint cells, several limitations should be acknowledged. First, while informative, the in vitro experiments using OUMS-27 cells do not fully mimic the complex physiological conditions of joint tissues, particularly those involving diseased chondrocytes and the ECM. Our horizontal co-culture system demonstrated that secreted EVs passed through the 600-nm filter and EVs were then incorporated into recipient cells. We need to confirm our findings using chondrocyte-derived EVs and/or synoviocyte-derived EVs. Moreover, the chondrocyte-synoviocyte interactions may differ significantly in a more complex tissue environment. Our model allowed us to monitor the direct incorporation of EVs into recipient cells using filters of different sizes, offering insights into the EV uptake for both chondrocytes and synoviocytes. We also need to examine in vivo (i.e., in the knee joint) considering that tissue complexity, including synovial fluid dynamics and mechanical stress, could modulate EV uptake in vivo. Therefore, future research should focus on exploring EV interactions with ECM components and assessing their behavior in the presence of multiple cell types and synovial fluid to gain a more comprehensive understanding of EV dynamics in joint tissues. In conclusion, our in vitro and in vivo findings confirm that EVs are effectively taken up by chondrocytes and synoviocytes. These vesicles function as critical mediators of communication within joint tissues and hold significant potential as cell-free therapeutic reagents for OA treatment. Further research into the mechanisms governing EV uptake and their therapeutic applications will be essential to advance EV-based therapies for joint diseases.

## 4. Materials and Methods

### 4.1. Reagents, Kits and Antibodies

For the EV marker assay, the Exosome Panel (ab275018; Abcam, Cambridge, UK) was used and CD9, Hsp70 and TSG101 were used as exosome marker from the Exosome Panel. Additional reagents used included PKH67 Fluorescent Cell Linker Kits (Sigma-Aldrich, Saint Louis, MO, USA), ExoSparkler EVs Membrane Labelling Kit-Red (EX02, Dojindo, Japan), the Cell Navigator F-Actin Labelling Kit (Green Fluorescence) (AAT Bioquest, Pleasanton, CA, USA) and dynasore (dynamin inhibitor I) (Calbiochem, Gibbstown, NJ, USA).

### 4.2. Preparation of Conditioned Medium and Isolation of Extracellular Vesicles

OUMS-27 cells were prepared following previously established protocols [36,37]. Briefly, the cells were seeded at a density of 1.0 × 10^6^ cells per dish (100 mm in diameter) and incubated in Dulbecco’s modified Eagle’s medium (DMEM; FUJIFILM Wako Pure Chemical Corporation, Osaka, Japan) containing 10% fetal bovine serum (FBS). Cells were passaged at rates of 1:2 to 1:4 by using trypsin with ethylenediaminetetraacetic acid. Cells at passage 3–9 were used for the experiments. After washing twice with PBS, the medium was replaced with FBS-free medium, and the cells were cultured for an additional two days. The conditioned medium was collected and subjected to sequential centrifugation: 15 min at 2000× *g* at 4 °C, followed by 60 min at 10,000× *g* at 4 °C, and then ultracentrifugation for 70 min at 100,000× *g* at 4 °C. The resulting pellet was resuspended in PBS and ultracentrifuged again for 70 min at 100,000× *g* at 4 °C to eliminate any remaining contaminants.

### 4.3. Size Distribution

NTA was performed using a NanoSight NS300 instrument (Malvern Instruments, Malvern, UK) at Kyoto University to measure particle size distribution in the conditioned medium. EVs were diluted in PBS and pumped into the flow cell. The particles were recorded by a camera and analyzed using NTA version 3.0 software. Settings included a camera level of 15, detection threshold of 5. For each sample, five 60-s recordings were taken.

### 4.4. Transmission Electron Microscopy

For TEM, a 400-mesh copper grid coated with formvar/carbon films was hydrophilically treated. The EV pellet, collected from 30 mL of conditioned medium, was resuspended in 30 μL of PBS. A 5-μL aliquot of the suspension was placed onto parafilm, and the grid was floated on the droplet for 15 min. The sample was then negatively stained with 2% uranyl acetate solution for 2 min. EVs were visualized at 20,000× magnification using an H-7650 transmission electron microscope (Hitachi, Tokyo, Japan) at the Central Research Laboratory, Okayama University Medical School.

### 4.5. Western Blotting Analysis

EVs were isolated from OUMS-27 cells following the previously described protocol. The EVs were lysed using CelLytic M Mammalian Cell Lysis/Extraction Reagent (Sigma-Aldrich) with the addition of proteinase inhibitors (Roche, Basel, Switzerland) to prevent protein degradation. Protein concentration was determined using the Pierce BCA Protein Assay kit (Thermo Fisher Scientific, Waltham, MA, USA) according to the manufacturer’s instructions. Western blotting analysis was carried out according to methods previously reported by our group [38,39]. Briefly, 10 μg of EV lysate was used for western blotting analysis. The primary antibodies were anti-CD9 (1:10,000), anti-Hsp70 (1:10,000) and anti-TSG101 (1:10,000). Secondary antibodies against anti-rabbit IgG (1:5000) were employed and developed using Amersham ECL Prime (GE Healthcare, Buckinghamshire, UK). All experiments were independently repeated at least three times.

### 4.6. Plasmid DNA Construction

Human CD9 (hCD9) cDNA was synthesized using a cDNA pool reverse-transcribed from mRNAs extracted from HeLa cells. The hCD9 cDNA fragment was amplified using the following primers: forward: 5′-GGAATTCCATATGCCGGTCAAAGGAGGCACCAAGTG-3′; reverse: 5′-CCCAAGCTTCTAGACCATCTCGCGGTTCC-3′. This fragment was then inserted into the NdeI/HindIII site of the pURE1 vector (Cosmo Bio, Tokyo, Japan), resulting in the construct pURE1-hCD9. To create the pCMV-tagRFP-hCD9 construct, a tag RFP-GGGSGGGS fragment and a GGGSGGGS-hCD9 fragment were amplified from template DNAs and primers. The tag RFP-GGGSGGGS fragment was generated using the template DNA pTagRFP-C (Evrogen, Moscow, Russia) with the following primers: forward: 5′-CTAGCTAGCATGGTGTCTAAGGGCGAAGAGCTGA-3′; reverse: 5′-CGACCCACCTCCGCCCGAGCCTCCGCCACCATTAAGTTTGTGCCCCAGTTTGCTAGGGAGGTC-3′. The GGGSGGGS-hCD9 fragment was obtained using the template DNA pURE1-hCD9 with the following primers: forward: 5′-GGTGGCGGAGGCTCGGGCGGAGGTGGGTCGCCGGTCAAAGGAGGCACCAAGTG-3′, reverse: 5′-GATATCCTAGACCATCTCGCGGTTCC-3′. The two fragments were annealed to obtain the tagRFP-hCD9 fragment, which was then inserted into the NheI/HindIII site of the pcDNA3.1 (+) expression vector. All plasmids were transfected into Escherichia coli strain DH5α for amplification, and the plasmid DNA was purified using the PureLink HiPure Plasmid Midiprep Kit (Thermo Fisher Scientific).

### 4.7. Stable Transgene Expression

HEK-293 cells were obtained from the Japanese Cancer Research Resource Bank Cell Bank (National Institute of Biomedical Innovation, Health and Nutrition, Osaka, Japan) and cultured in MEM medium (Gibco, Rockville, MD, USA) supplemented with 10% heat-inactivated FBS and penicillin/streptomycin at 37 °C in 5% CO_2_, as previously described [40]. HEK-293 cells were seeded in 24-well plates and cultured for 24 h. The cells were then transfected with pCMV-tagRFP-hCD9 using Lipofectamine 2000, following the manufacturer’s instructions. The medium was replaced with fresh medium 4 h post-transfection. After 24 h, the medium was replaced with G418 medium (1200 µg/mL, Nacalai Tesque, Kyoto, Japan) to select polyclonal HEK-293-tagRFP-hCD9-transduced cells.

### 4.8. Chondrocytes

Cartilage tissues were minced and digested with pronase (FUJIFILM Wako Pure Chemical Corporation) for 30 min at 37 °C, followed by collagenase digestion for 18–24 h at 37 °C with gentle agitation [41]. Tissue debris was removed using a cell strainer, and the cells were washed twice with DMEM supplemented with 10% 4-(2-hydroxyethyl)-1-piperazineethanesulfonic acid (HEPES, Life Technologies, Tokyo, Japan), 100 IU/mL of penicillin, and 100 mg/mL of streptomycin. The isolated cells were seeded into 24-well microtiter plates (Costar, Cambridge, MA, USA) at a density of 2 × 10^6^ cells/mL in 2 mL of DMEM supplemented with 10% HEPES, 100 IU/mL of penicillin, and 100 mg/mL of streptomycin. The plates were incubated at 37 °C in a humidified atmosphere containing 5% CO_2_, with chondrocyte cultures passaged weekly until primary cultures reached confluence.

### 4.9. Synoviocytes

Synovial tissues were minced and digested with collagenase (FUJIFILM Wako Pure Chemical Corporation) and DNase I (Sigma-Aldrich) at 37 °C. The tissue debris was removed using a cell strainer, and the cells were washed twice with DMEM supplemented with 10% HEPES, 100 IU/mL of penicillin, and 100 mg/mL of streptomycin. The isolated cells were seeded into 24-well microtiter plates at a density of 2 × 10^6^ cells/mL in 2 mL of DMEM supplemented with 10% HEPES, 100 IU/mL of penicillin, and 100 mg/mL of streptomycin. The plates were incubated at 37 °C in a humidified atmosphere containing 5% CO_2_. Synovial tissue cultures were passaged weekly until the third passage, where the cells exhibited a homogeneous fibroblast-like morphology [42].

### 4.10. Interactive Co-Culture

An interactive co-culture system (NICO-1; Ginrei Lab, Ishikawa, Japan) was employed to analyze EV production and transfer. The co-culture system was incubated at 37 °C in a humidified atmosphere containing 5% CO_2_. This system connects two chambers horizontally, allowing two cell lines to be seeded in each chamber while sharing the culture medium but being separated by a pore filter. EVs secreted from the cells diffuse through the medium, with some passing through the pores. RFP-CD9 HEK-293 cells (7.0 × 10^4^) and OUMS-27 cells (5.0 × 10^4^) were seeded separately using a 30-nm filter (Figure 4a,e). The same cells were also seeded with a 600-nm filter (Figure 4b,f). Additionally, RFP-CD9 HEK-293 cells (7.0 × 10^4^) were co-cultured with chondrocytes from patients with OA (5.0 × 10^4^) and synoviocytes (5.0 × 10^4^) using a 600-nm filter (Figure 4c,d,g,h). The co-cultures were maintained for 4 days, then washed twice with PBS, fixed with 4% paraformaldehyde for 10 min, washed twice with PBS again, and stained with Hoechst 33258 (1:5000) and iFluor 488 Phalloidin (1:1000) for 1 h at 20 °C. Images were captured using a BZ-X810 microscope (KEYENCE, Osaka, Japan).

### 4.11. EVs Labelling with PKH67 and Uptake by OUMS-27 Cells

EVs were isolated from OUMS-27-conditioned medium via ultracentrifugation and labelled with PKH67 using the PKH67 Fluorescent Cell Linker Kit according to the manufacturer’s protocol, with minor modifications. Briefly, 500 μL of 0.4% PKH67/Diluent C was added to 200 μL of the EV suspension, followed by the addition of 1200 μL of 1% bovine serum albumin (filtered through a 0.22 μm membrane) to absorb excess PKH67. The suspension was washed through a 100 kDa pore filter with 1 mL of PBS three times and washed twice with DMEM in the absence of FBS. Centrifugation was performed at 4 °C at 10,000× *g* for 5 min. The EVs were washed with 0.5 mL of PBS three times, then resuspended in 25 μL of PBS. OUMS-27 cells (1.5 × 10^4^) were seeded onto 0.1% type I collagen-coated glass with FlexiPERM eight-well grids (Zarstedt, Nümbrech, Germany) for 2 days. Cells were then cultured with 3.0 × 10^7^ particles of PKH67 labelled EVs for 3 h, 6 h, 12 h and 24 h. Images of the labelled EVs were captured and analyzed using a BZ-X810 microscope.

### 4.12. EV Uptake Inhibition

OUMS-27 cells (1.5 × 10^4^) were seeded on glass coverslips coated with 0.1% type I collagen using flexiPERM eight-well grids. After 2 days, the medium was removed and the cells were washed twice with PBS. The cells were then incubated in serum-free medium containing 20 µM dynasore or same concentration of vehicle for 24 h. Following incubation, the cells were treated with 3.0 × 10^7^ PKH67-labelled EV particles for an additional 24 h. Images of the labelled EVs were captured and analyzed using a BZ-X810 microscope system [43].

### 4.13. EVs Labelling and Injection into Mice Knee Joints

EVs isolated from OUMS-27 cells were labelled with Mem Dye-Deep Red using the ExoSparkler Exosome Membrane Labelling Kit following ultracentrifugation. C57BL/6J mice (10 weeks old, 22–28 g) were obtained from the Department of Animal Resources at the Okayama University Advanced Science Research Center. Under anesthesia, 3.0 × 10^8^ Mem Dye-Deep Red-labelled EV particles in 20 μL of PBS were injected into the left hind leg joint cavity of each mouse. After 24 h, the mice were sacrificed. Images of the labelled EVs were captured and analyzed using a BZ-X810 microscope.

### 4.14. Kawamoto Method

Frozen sections of joint tissue samples were prepared according to Kawamoto’s film method [44]. Briefly, the mice were euthanized by cervical dislocation and immediately fixed with 4% paraformaldehyde to terminate the hypoxic reaction. The knees were then excised, frozen in hexane with dry ice, and embedded in SCEM compound (Section Lab, Hiroshima, Japan). Joint tissues were sectioned sagittally with 5-μm thickness using Cryofilm Type IIC (Section-lab) on a Leica cryostat. Nuclei were stained with Hoechst 33258 according to the standard protocol. Images of the labelled EVs were captured and analyzed using a BZ-X810 microscope [43].

## Data Availability

All data generated or analyzed in this study are included in the article.
